# Scientific publications and the curriculum vitae: A medical student's Achilles' heel?

**DOI:** 10.3126/nje.v7i1.17756

**Published:** 2017-03-31

**Authors:** Brijesh Sathian, Huda Fatima, Syed Ather Hussain, Ritesh G Menezes

**Affiliations:** 1 Department of Community Medicine, Manipal College of Medical Sciences, Pokhara Nepal; 2 Dow Medical College, Dow University of Health Sciences, Karachi Pakistan; 3 Forensic Medicine Division, Department of Pathology, College of Medicine King Fahd Hospital of the University, University of Dammam, Dammam, Saudi Arabia

With the number of hopeful students eager to surge ahead in the noble profession of medicine, increasing every day, the world of both academic as well as clinical medicine is fast becoming more competitive. Thanks to globalization, our world is far more interconnected than it ever was. Discoveries and breakthroughs are occurring right this minute and are being transmitted live the very next. This is an exciting time to be a part of the medical community. There is no limit, no barrier to what a person may achieve, albeit with untiring hard work and an unflagging determination.

But each barrier breaking record becomes a double-edged sword for the aspiring doctor - on the heels of reading about such accomplishments and achieved feats, comes the realization that one must aim to go further than that, if he/she wishes to leave his/her indelible footprints on the sands of his/her chosen career.

Most medical students realize early on in their years of undergraduate education that they face a vast expanse of uncertain, rocky and treacherous terrain that must be traversed before one can safely plant a triumphant flag on the other side and be counted amongst the successful physicians of today. This arduous journey extends beyond just completing five years of medical school - it further consists of mind numbing exams and brutally demanding interviews and demonstrations of his/her clinical skills and knowledge, and it would not be incorrect to say that a medical student's faithful companion throughout his/her journey in these stormy, turbulent seas is his/her curriculum vitae (CV).

However, a CV can be much more than a mere companion- if worked upon, it can be the Holy Grail that serves to unlock many doors for the aspiring physician. It is no wonder that a well compiled CV showcasing the fruit of one's persistent hard work of many years is of almost as much importance as a student's examination scores. A CV is the silent introduction, even before the medical student enters through the door. It speaks of his/her dedication to his/her chosen profession; and nothing speaks more highly of a potential doctor than his/her publications as they display his/her commitment to research.

There can be no denying the importance of scientific publications in any academic's life. But one might argue whether a student who hopes to be practicing clinical medicine some day, should feel the need to exhibit several publications in his/her CV, merely in hope of gaining an edge over his/her competitors. Suffice to say that in today's cut-throat world, one can leave no stone unturned when applying for a residency and competing against fellow medical students and the concept of 'publish or perish' has permeated their collective conscious, even before their careers have taken off. This then begs the question - how much is too much?

Which is better, having a greater number of publications on one's CV or perhaps a few well selected, chosen ones? A large number of publications demonstrates a student's hard work and aptitude; they show his/her consistency and his/her steady, continuous dedication to furthering his/her knowledge and honing his/her intellectual capabilities. It further expresses his/her enthusiasm to course ahead in medicine and may well impress a future employer by communicating that the aforesaid individual is an all rounder, with better time management, one who keeps abreast with current medical research despite time constraints and would be able to think outside the box, or medical textbooks in this case.

On the other hand, fewer yet well composed publications in leading peer-reviewed journals, which explain an author's germinating idea well enough to be utilized as a foundation stone for future researchers, are no doubt more invaluable to the cause of medical research at the end of the day. If such a publication is present on a medical professional's CV, it would mean that the author has a genuinely keen interest in pursuing research rather than subscribing to the popular 'bitesize science' attitude that many of his/her peers are currently displaying towards research. The pressure to get published takes away the charm of trying to improve clinical and community medicine via scientific research and thus many medical students and professionals resort to publishing relatively less useful articles, just to increase the quantity of their publications. This brings us to another aspect of scientific publications and the CV: considering that a prospective candidate would wish to flaunt his strengths at a glance, would it make more sense for publications to be categorized as PubMed-indexed versus non PubMed-indexed on a CV? While a PubMed-indexed journal article publication would definitely shine a light upon a student's merit and worth, one must weigh the importance in comparison to the overall value of the publication in question. Just because a research has not been published in a PubMed-indexed journal, does not necessarily translate to it being less meaningful.

Similarly, should the ‘impact factor’ of the journal where the article is published be mentioned on a medical student's CV? Once again, it would add to the credence of the future doctor if he/she has managed to publish in the notable giants of today's medical journals, for example, The Lancet, which has a current impact factor of 45.217 and is one of the world's oldest and leading medical journals.

Lastly, another leading question amongst medical students is the importance of the authorship position and whether this should be mentioned on one's CV. If one has multiple publications, then should the CV have segregated sections for publications in which the applicant is the first author and those in which his/her authorship position is further down the list? There can be no denying that the growing complexity of research as well as the increasing connectivity in today's world has led to an increase in the number of collaborators on publications, but having first authorship is a fairly accurate estimate that the person in question has done the lion's share of the research work [1 ]. 

While such questions can be answered best by program directors and those in charge of interviewing and selecting applicants, there can be no doubt that mentioning all of the above in one's CV would appear to give the student an edge over his/her competitors. However, we must realize the enormous burden placed upon these young professionals’ shoulders, which might even lead to dishonest and fraudulent publication practices, primarily because medical students are at the very start of their academic and professional life, so they have more to gain and not much to lose by underhanded attempts to secure publications on their CV [ 2].That being said, however, the life of a doctor is undoubtedly one of lifelong hard work and learning, which medical students do not shy away from. Therefore, the sooner these queries regarding the importance of scientific publications on a CV are clarified and the cloud of confusion is cleared, the smoother the road to success will become. 
